# The National Centre for Healthy Ageing data platform: establishing an Electronic Health Record derived linked geographic cohort.

**DOI:** 10.23889/ijpds.v7i3.1949

**Published:** 2022-08-25

**Authors:** Nadine Andrew, Richard Beare, Tanya Ravipati, Emily Parker, Taya Collyer, David Ung, Velandai Srikanth

**Affiliations:** 1 National Centre for Healthy Ageing and Peninsula Clinical School, Central Clinical School, Monash University; 2 National Centre for Healthy Ageing and Peninsula Health

## Objectives

Electronic Health Record (EHR) data have created unique opportunities for research. However, these data are: not curated, siloed and poorly integrated. We describe linkage of EHR data from an entire health service with government datasets to establish a linked geographic cohort within the Australian National Centre for Healthy Ageing (NCHA).

## Approach

Research suitable EHR items were identified from Peninsula Health (NCHA partner) data systems based on: published research, availability and quality. Items underwent end-user Delphi processes to identify core research items (consensus=70%). Approvals were obtained from the Australian Institute of Health and Welfare (AIHW) for linkage with: Medicare, medication dispensings, Aged Care and death registry data through the AIHW spine, created using identifiers from the Medicare Consumer Directory (MCD); and from the Centre for Victorian Data Linkage for linkage to state-wide hospital data. Identifiers for local residents aged ≥60 years who attended Peninsula Health were submitted for probabilistic data linkage.

## Results

Delphi participants included 10 researchers from 8 fields/departments and 13 clinicians from 11 clinical areas. To date 7 of the 11 datasets have been reviewed. N=107 potentially suitable data items were identified and 96 gained consensus for inclusion in the core dataset. Of the 49,767 Health Service users (episodes: Jan 2010-Dec May 2021) submitted for linkage, 98.4% were successfully linked to the MCD (Median age 72.2 years, 52.2% female, 1.8% regional residence). An additional 172,290 individuals living within the geographic region but not contained within the EHR dataset were identified in the MCD for linkage to the government datasets. Linkage accuracy was impacted by inaccurate/incomplete address fields (~30%) and lack of adherence to naming conventions within the EHR data.

## Conclusion

Linking with EHR data is complex. Having an established EHR research dataset will improve the feasibility of data linkage and potential for future expansion of linkages within the NCHA. Once merged, the data will be used to underpin a range of research activities related to ageing and dementia.

